# Analysis of acquired resistance mechanisms to osimertinib in patients with EGFR-mutated advanced non-small cell lung cancer from the AURA3 trial

**DOI:** 10.1038/s41467-023-35962-x

**Published:** 2023-02-27

**Authors:** Juliann Chmielecki, Tony Mok, Yi-Long Wu, Ji-Youn Han, Myung-Ju Ahn, Suresh S. Ramalingam, Thomas John, Isamu Okamoto, James Chih-Hsin Yang, Frances A. Shepherd, Krishna C. Bulusu, Gianluca Laus, Barbara Collins, J. Carl Barrett, Ryan J. Hartmaier, Vassiliki Papadimitrakopoulou

**Affiliations:** 1 Translational Medicine, Oncology R&D, AstraZeneca, Boston, MA USA; 2 State Key Laboratory of Translational Oncology, Department of Clinical Oncology, Chinese University of Hong Kong, Hong Kong, China; 3 Guangdong Lung Cancer Institute, Guangdong Provincial People’s Hospital & Guangdong Academy of Medical Sciences, Guangzhou, China; 4 Center for Lung Cancer, National Cancer Center, Goyang, Republic of Korea; 5 Section of Hematology-Oncology, Samsung Medical Center, Sungkyunkwan University School of Medicine, Seoul, Republic of Korea; 6 Department of Hematology and Medical Oncology, Winship Cancer Institute, Emory University School of Medicine, Atlanta, GA USA; 7 Medical Oncology, Olivia Newton-John Cancer Research Institute, Austin Health, Melbourne, VIC Australia; 8 Department of Respiratory Medicine, Graduate School of Medical Sciences, Kyushu University, Fukuoka, Japan; 9 Department of Medical Oncology, National Taiwan University Cancer Center, Taipei, Taiwan; 10 Departments of Medical Oncology and Hematology, Princess Margaret Cancer Centre, and the University of Toronto, Toronto, ON Canada; 11 Translational Medicine, Oncology R&D, AstraZeneca, Cambridge, UK; 12 Biometrics and Information Sciences, AstraZeneca, Cambridge, UK; 13 Department of Thoracic/Head and Neck Medical Oncology, University of Texas M.D. Anderson Cancer Center, Houston, TX USA; 14 Present Address: Clinical Development, Merus, Utrecht, The Netherlands; 15 Present Address: Simbiotic Consulting Ltd, Glasgow, UK; 16 Present Address: Clinical Development, Pfizer Inc, Houston, TX USA

**Keywords:** Cancer, Lung cancer, Targeted therapies

## Abstract

Osimertinib, an epidermal growth factor receptor tyrosine kinase inhibitor (EGFR-TKI), potently and selectively inhibits EGFR-TKI-sensitizing and *EGFR* T790M resistance mutations. This analysis evaluates acquired resistance mechanisms to second-line osimertinib (n = 78) in patients with *EGFR* T790M advanced non-small cell lung cancer (NSCLC) from AURA3 (NCT02151981), a randomized phase 3 study comparing osimertinib with chemotherapy. Plasma samples collected at baseline and disease progression/treatment discontinuation are analyzed using next-generation sequencing. Half (50%) of patients have undetectable plasma *EGFR* T790M at disease progression and/or treatment discontinuation. Fifteen patients (19%) have >1 resistance-related genomic alteration; *MET* amplification (14/78, 18%) and *EGFR* C797X mutation (14/78, 18%).

## Abstract (web summary)

In the phase III AURA3 study (NCT02151981), the third-generation epidermal growth factor receptor tyrosine kinase inhibitor osimertinib prolonged progression-free survival versus platinum-doublet chemotherapy in patients with EGFR T790M advanced NSCLC. Here, by next-generation sequencing of circulating tumor DNA, the authors assess candidate mechanisms of acquired resistance to osimertinib in patients from the AURA3 trial.

## Introduction

Epidermal growth factor receptor tyrosine kinase inhibitors (EGFR-TKIs) are effective treatments for patients with advanced non-small cell lung cancer (NSCLC) harboring an EGFR-TKI sensitizing mutation (EGFRm)^1^. Most patients who are treated with EGFR-TKIs develop resistance, with 50–60% of patients whose disease progresses whilst receiving first- and second-generation EGFR-TKIs harboring the *EGFR* T790M mutation^2–6^.

Osimertinib is a third-generation, irreversible, oral EGFR-TKI that potently and selectively inhibits both EGFRm and *EGFR* T790M resistance mutations^7,8^. It is approved as a first-line treatment for patients with EGFRm advanced NSCLC and for patients with *EGFR* T790M advanced NSCLC following progression on an EGFR-TKI, and has efficacy in patients with NSCLC central nervous system (CNS) metastases^9–11^. Approval for patients with *EGFR* T790M advanced NSCLC is based on the Phase III AURA3 study (NCT02151981). In this study, osimertinib significantly prolonged progression-free survival (PFS) (median 10.1 versus 4.4 months; hazard ratio (HR] 0.30 (95% confidence interval (CI) 0.23 to 0.41]; *P* < 0.001) and improved objective response rate (ORR; 71% versus 31%) versus platinum-doublet chemotherapy in patients with *EGFR* T790M advanced NSCLC, following disease progression on first-line EGFR-TKI therapy^7^. The final analysis for overall survival (OS) did not show a statistically significant benefit with osimertinib versus platinum-doublet chemotherapy (median 26.8 versus 22.5 months; HR 0.87 (95% CI 0.67 to 1.12; *P* = 0.277); however, there was a high crossover rate from chemotherapy to osimertinib^12^.

A number of small-scale studies have reported candidate resistance mechanisms to osimertinib at the point of disease progression when it is used as a second- or later-line treatment^13–16^, and more recently, when used in the first-line setting, including from the Phase III FLAURA study^17,18^. Functional studies for many pathways of acquired resistance to osimertinib have been reported previously^19–21^. However, understanding resistance mechanisms is important to help define appropriate combination therapies for patients with EGFRm advanced NSCLC following acquired resistance to EGFR-TKIs or to prevent the development of resistance. Tumor-specific molecular characteristics can be tested using circulating tumor DNA (ctDNA) isolated from the plasma of some cancer patients, thus providing a potentially valuable biomarker status that can be obtained in a minimally invasive manner^22–24^. The data available for resistance mechanisms to osimertinib are limited and collected from across different studies using diverse methodologies, with the majority of resistance studies focusing on ctDNA versus tissue analysis^14,16,21^. A heterogenous mixture of resistance mechanisms to osimertinib have been detected including *EGFR* mutations and *MET* amplification^13,14,16^. A loss of detectable T790M has also been reported in 42–68% of patients^13,14,21^. Here we report data on the plasma ctDNA genomic profile of patients with *EGFR* T790M advanced NSCLC, whose disease progressed on second-line osimertinib treatment during the Phase III AURA3 study.

## Results

### Demographics

**Fig. 1:**
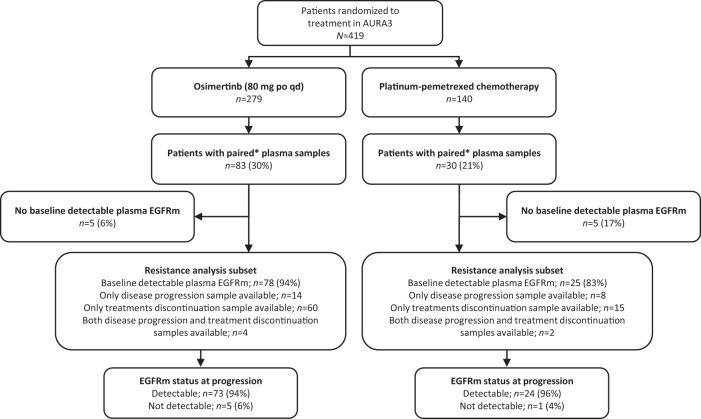
Patient disposition. CONSORT flow diagram of patient disposition and eligibility in the analysis of mechanisms of acquired resistance in the AURA3 trial. *Plasma provided at baseline and at disease progression or treatment discontinuation. EGFR epidermal growth factor receptor, p.o, orally, qd once daily, TKI tyrosine kinase inhibitor.

In AURA3, 279 patients were randomized to osimertinib and 140 to platinum-pemetrexed; 83 (30%) and 30 (21%), respectively, had paired (baseline sample and sample at disease progression and/or treatment discontinuation) plasma samples analyzed by NGS (Fig. 1). Among patients with treatment discontinuation samples, 54 (84%) in the osimertinib arm and 16 (94%) in the platinum-pemetrexed arm discontinued due to disease progression. Other reasons for treatment discontinuation included adverse event (*n* = 5 [8%]), subject decision (*n* = 2 [3%]), and other (*n* = 3 [5%]) in the osimertinib arm, and maximum cycle of chemotherapy reached (*n* = 1 [6%]) in the platinum-pemetrexed arm.

Among patients with paired plasma samples, 103/113 (91%) had baseline detectable plasma *EGFR* mutations (Ex19del/L858R and/or T790M), and were included in the acquired resistance analysis subset: 78/83 (94%) in the osimertinib arm and 25/30 (83%) in the platinum-pemetrexed arm (Fig. 1). Within this subset, most patients (75/103, 73%) had plasma samples only available at treatment discontinuation, compared with patients with samples only available at disease progression (22/103, 21%). A small number of patients had samples available at both disease progression and treatment discontinuation (6/103; 6%); results from both time points were in the analysis for these patients.

Generally, baseline demographics and clinical characteristics for patients included in the resistance analysis subset were consistent with those reported for all patients randomized in AURA3; baseline demographics for the osimertinib arm are shown in Table 1. Slightly more patients in the osimertinib arm received prior treatment with erlotinib and slightly fewer received gefitinib compared with all patients randomized to osimertinib.

**Table 1:** Baseline characteristic of osimertinib-treated patients evaluable for analysis of acquired resistance mechanisms

Characteristic	AURA3 intent-to-treat population (*n* = 279)^7^	Subset with valid NGS results* (*n* = 83)	Evaluable for resistance analysis subset *(n* = 78)
Median age (range), yr	62 (25–85)	61 (25–82)	61 (25–82)
Female sex, no. (%)	172 (62)	49 (59)	45 (58)
Race, no. (%)^†^			
White	89 (32)	23 (28)	23 (29)
Asian	182 (65)	59 (71)	54 (69)
Other	8 (3)	1 (1)	1 (1)
No history of smoking, no. (%)	189 (68)	56 (67)	52 (67)
Disease classification, no. (%)			
Adenocarcinoma histology not otherwise specified	232 (83)	69 (83)	65 (83)
Metastatic disease	266 (95)	81 (98)	76 (97)
CNS metastases^‡^	93 (33)	27 (33)	26 (33)
Extrathoratic visceral metastases	145 (52)	50 (60)	47 (60)
*EGFR* mutation type			
T790M	275 (99)	82 (99)	77 (99)
L858R	83 (30)	23 (28)	20 (26)
G719X	4 (1)	1 (1)	1 (1)
S768I	1 (<1)	0	0
Exon 19 deletion	191 (68)	59 (71)	57 (73)
Exon 20 insertion	1 (<1)	0	0
Previous EGFR-TKI therapy			
Gefitinib	166 (59)	44 (53)	39 (50)
Erlotinib	96 (34)	34 (41)	34 (44)
Afatinib	20 (7)	6 (7)	6 (8)

^*^Patients with paired plasma samples that had valid NGS results at baseline and at the time of disease progression or treatment discontinuation.

^†^Race was self-reported. The category of “other” includes black, American Indian, and Alaska Native

^‡^CNS metastases were determined programmatically from baseline data of CNS lesion site, medical history, and/or surgery, and/or radiotherapy. The patient was identified as having a locally advanced disease in the brain.

CNS central nervous system, EGFR-TKI epidermal growth factor receptor tyrosine kinase inhibitor, NGS next-generation sequencing, WHO World Health Organization.

### Acquired resistance mechanisms by treatment arm (plasma ctDNA analysis): Osimertinib arm

**Fig. 2:**
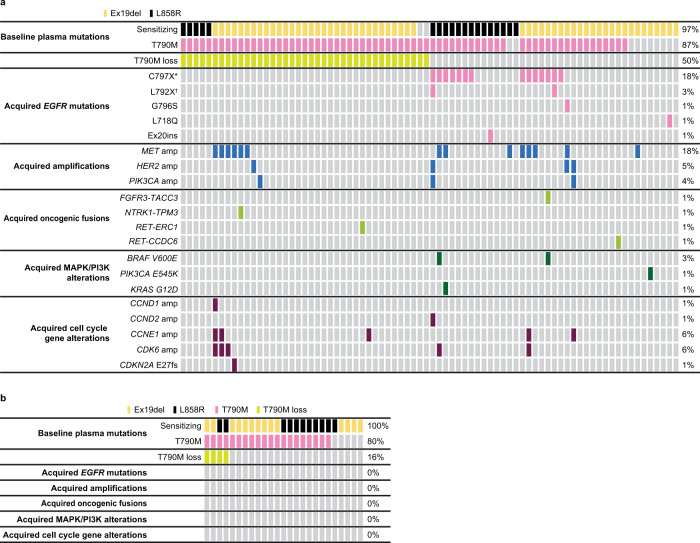
Acquired alterations in osimertinib-treated patients and in chemotherapy-treated patients. Tile plots indicating **A** acquired alterations in osimertinib-treated patients (*n* = 78) and **B** acquired alterations in chemotherapy-treated patients (*n* = 25) from the AURA3 trial. Source data are provided in the Supplementary Data 1 file. *C797S or C797G; ^†^L792F or L792H. EGFR epidermal growth factor receptor.

In the osimertinib arm acquired resistance analysis subset, 32/78 (41%) patients had at least one detectable acquired resistance mechanism; 46 (58%) had no detectable candidate mechanism of resistance (Fig. 2A). *EGFR* mutations and *MET* amplification were the most common acquired resistance mechanisms detected, occurring in 17 (22%) and 14 (18%) patients, respectively. Acquired *HER2* amplification, MAPK/PI3K alterations and oncogenic fusions (*FGFR3-TACC3*, *NTRK1-TMP3*, *RET-ERC1,* and *RET-CCDC6*) were each detected in four patients (5%); *PIK3CA* amplification was detected in three patients (4%). Acquired *EGFR* mutations included C797X in 14 patients (18%), two patients with C797X co-occurring with L792X, and one patient each with G796S, L718Q, and exon 20 insertion. A total of 39 (50%) patients had a loss of detectable plasma *EGFR* T790M at progression and/or treatment discontinuation, of which 10/39 (26%) had acquired alterations (Fig. 2A). Ten patients (13%) had undetectable *EGFR* T790M at baseline. Among patients with *MET* amplification and detectable plasma *EGFR* T790M at baseline, 6/12 (50%) lost *EGFR* T790M detection at progression and/or treatment discontinuation (Fig. 2A). In contrast, none of the patients with acquired *EGFR* mutations and detectable baseline plasma *EGFR* T790M lost *EGFR* T790M detection at progression and/or treatment discontinuation.

More than one acquired resistance mechanism was detected in 15 (19%) patients (Fig. 2A), meaning 47% of all 32 patients with an acquired resistance mechanism had multiple mechanisms detected. *MET* amplification co-occurred with *EGFR* C797X (C797S or C797G) in five patients, including two patients with cell cycle gene alterations, one of whom also had *BRAF* V600E, and one patient with *KRAS* G12D. Further co-occurrence with *MET* amplification was detected in three other patients with cell cycle gene alterations, detected in one patient with *TPM3-NTRK1* fusion, and in one patient with *EGFR* G796S + *HER2* amplification. Two further patients with *HER2* amplification had co-occurring *PIK3CA* amplification and cell cycle gene alterations, including one patient with co-occurring *EGFR* C797G/L792H/L792F. One patient with *FGFR3-TACC3* fusion had co-occurring *EGFR* C797S + *BRAF* V600E.

Non-genomic mechanisms of resistance, such as small cell transformations may have been present, but could not be detected using NGS; histological assessment of tumor tissue at progression/treatment discontinuation was not available to compare these resistance mechanisms.

### Acquired resistance mechanisms by treatment arm (plasma ctDNA analysis)**:** platinum-pemetrexed arm

In the platinum-pemetrexed arm acquired resistance analysis subset (*n* = 25), acquired alterations included loss of amplification in *MET* (*n* = 2), *HER2* (*n* = 1), and *PIK3CA* (*n* = 1). One patient had a *PTEN* truncating mutation. There were no *EGFR* C797S mutations, no acquired mutations in *H/N/KRAS*, *BRAF*, *FGFR1*, *PIK3CA*, no acquired *MET*, *HER2*, or *FGFR1* amplifications, and no oncogenic fusions. In total, 4/25 patients (16%) lost detectable *EGFR* T790M at progression/treatment discontinuation; five patients (20%) had undetectable *EGFR* T790M at baseline (Fig. 2B).

### Osimertinib duration of treatment by candidate resistance mechanisms

**Fig. 3:**
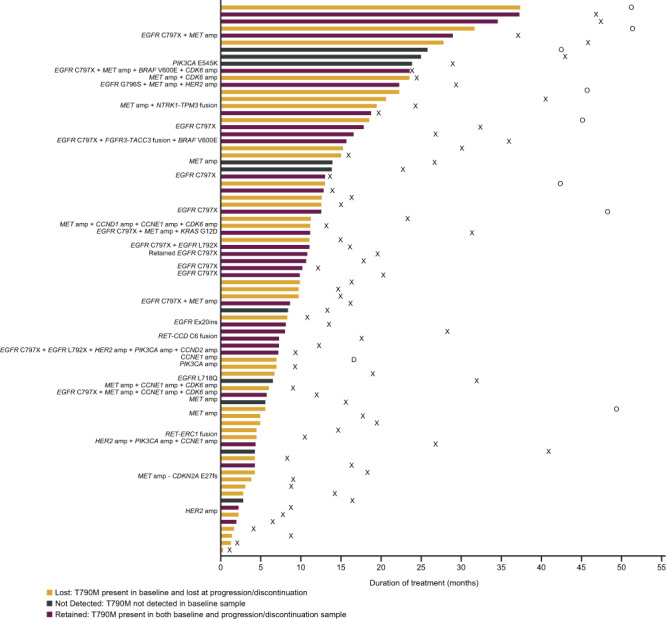
Osimertinib duration of treatment by candidate mechanism and T790M status. Swimmer plot indicating the duration of treatment with osimertinib (months) by candidate mechanism and T790M status (*n* = 78). Source data are provided in the Supplementary Data 1 file. X time of death for patients who have died, O date last known alive for patients who have not died, D time of study discontinuation.

Among patients in the osimertinib arm acquired resistance analysis subset (*n* = 78), duration of treatment was variable across patients with different acquired mechanisms of resistance and loss or retention of detectable *EGFR* T790M at progression (Fig. 3). Due to the heterogeneity of acquired resistance mechanisms, there was no clear correlation between type or co-occurrence of acquired mechanisms of resistance and duration of treatment.

## Discussion

The data presented in this exploratory analysis of the AURA3 study represent, to the best of our knowledge, the largest study of resistance mechanisms in a single cohort of patients with similar baseline characteristics from a randomized controlled study who received second-line osimertinib. Numerous acquired resistance mechanisms were detected with osimertinib, the most common being acquired *EGFR* mutations and *MET* amplification, occurring in 22% and 18% of patients, respectively. The most common *EGFR* mutation was C797S, reported in 14% of patients; less frequently reported *EGFR* mutations included, C797G, L792H/F, G796S, L718Q, and exon 20 insertion. Other resistance mechanisms observed with osimertinib included; *HER2* amplification, *PIK3CA* amplification, cell cycle gene alterations, and oncogenic fusions *FGFR3-TACC3*, *NTRK1-TPM3 RET-ERC1,* and *RET-CCDC6*.

The acquired resistance mechanisms detected in this study are consistent with previous reports for osimertinib in later-line settings, where *MET* amplification and *EGFR* C797S have also been reported as among the most common resistance mechanisms^14,16,17,21^. *EGFR* C797S was detected in 22–29% of patients in these studies, higher than the frequency reported here, while higher frequencies of up to 50% have been reported for acquired *MET* amplification. It should be noted that there is no consensus on the criteria for defining *MET* amplification with NGS. Furthermore, *MET* polysomy cannot be effectively accounted for with NGS^25^. A similar pattern of acquired resistance mechanisms has been reported following first-line osimertinib treatment in the phase 3 FLAURA study^18,26^ and in a recent large study comparing acquired resistance between first- and second/later-line osimertinib^17^. There is a degree of overlap between the mechanisms of resistance reported here and after treatment with first- and second-generation EGFR-TKIs. Although *EGFR* T790M is the most common mechanism of resistance following first- or second-generation EGFR-TKIs, amplification of *EGFR, MET*, *HER2,* and *PIK3CA* mutations have also been identified^3,27^. Of note, no new resistance mechanisms were identified in this study with second-line osimertinib that could lead to a more aggressive disease.

Loss of detectable *EGFR* T790M was reported in approximately half of the AURA3 patients studied here and in these patients, no clear association with a shorter duration of treatment was observed compared with patients who retained *EGFR* T790M. Although the possibility that *EGFR* T790M was undetected due to the assay’s limit of detection cannot be discounted, the baseline EGFR-TKI sensitizing mutation was detectable in the majority of patients at progression, indicating sufficient levels of ctDNA. Approximately a third of those patients with a loss of detectable *EGFR* T790M had at least one detectable acquired resistance mechanism, including activation of pathways either downstream or parallel to *EGFR*, including *MET*, *HER2,* and *PIK3CA* amplifications or cell cycle gene alterations. In a previous analysis of patients with T790M NSCLC and acquired resistance to osimertinib, patients with loss of detectable *EGFR* T790M had a shorter median time to treatment discontinuation compared with patients who retained *EGFR* T790M (6.1 months versus 15.2 months)^14^.

Co-occurrence of acquired resistance mechanisms was common in this study and could have clinical implications when determining subsequent treatments, highlighting the need for combination therapies to overcome multiple resistance mechanisms; for example, *MET* amplication co-occurred with *HER2* amplification and *NTRK* fusions. Data from the phase 1b TATTON study (NCT02143466) provided early evidence for the combination of osimertinib and savolitinib, a MET inhibitor, in patients with *MET*-amplified advanced NSCLC who progressed after receiving ≥1 first-, second-, or third-generation EGFR-TKIs (median duration of response 7.1 months, objective response rate (ORR] 52%)^28,29^, highlighting the importance of maintaining EGFR inhibition in subsequent lines of therapy. Another study addressing EGFR and MET inihibiton was the Phase Ib/II study of capmatinib plus gefitinib, which demonstrated preliminary clinical activity (ORR 47%) in patients with *EGFR*-mutated NSCLC and *MET*-amplified tumors after progression on EGFR-TKI therapy^10^.

To address the acquired *EGFR* C797S mutation, a potential therapeutic option could be to combine osimertinib with a first-generation EGFR-TKI which does not require *EGFR* C797 for activity. The ongoing ORCHARD platform study is investigating osimertinib plus gefitinib in patients with acquired *EGFR* C797X following progression on first-line osimertinib^30^. In addition, preclinical data have suggested that osimertinib combined with gefitinib, which is active against *EGFR* C797S, may delay the emergence of acquired resistance^31^. Preliminary clinical evidence also supports the potential for this combination, as demonstrated in an ongoing Phase I/II study where concurrent osimertinib plus gefitinib for the first-line treatment of *EGFR*-mutated NSCLC resulted in an ORR of 85% with rapid plasma clearance of the *EGFR* mutation^32^.

For other acquired mutations, interesting results have also been obtained in preclinical studies. For example, co-treating tumor cells that are *EGFR* T790M and *BRAF* V600E positive with osimertinib and the *BRAF* V600E inhibitor encorafenib increased tumor sensitivity compared with encorafenib treatment alone^33^. Further research is needed to elucidate the mechanisms of resistance to second-line osimertinib, and the therapeutic strategies to address them.

Caution should be taken when interpreting these data due to the exploratory nature of this analysis. Amplification events may be underestimated due to a high false-negative rate of plasma NGS for amplification compared with fluorescent in situ hybridization (FISH)^25^. As plasma NGS only detects genomic alterations in ctDNA, other non-genomic mechanisms of resistance including histological transformation (e.g., small cell lung cancer [SCLC]) were not evaluated, though it would be possible to study these potential mechanisms of resistance in the future trials that involve tissue samples^17^. Additionally, as no paired tissue biopsies were available for analysis, the plasma mutations could not be compared with tissue.

In conclusion, multiple mechanisms of resistance to second-line osimertinib were observed, similar to those observed in previous studies, with no predominating single mechanism identified. The most frequent resistance mechanisms were *MET* amplification and the *EGFR* C797S mutation and approximately half of the patients had a loss of detectable *EGFR* T790M. Importantly, no new mutations that lead to more aggressive cancer biology were detected. The results identify the need for tissue samples to be taken in order to further investigate non-genomic mechanisms of resistance including histological transformation and for continued investigation into combination therapy approaches to prevent or overcome emergent resistance.

## Methods

### Standard protocol approvals, registration, and patient consent

The study was approved by the institutional review board/independent ethics committee associated with each study center. This study was performed in accordance with the ethical principles that have their origin in the Declaration of Helsinki and that are consistent with International Conference on Harmonization/Good Clinical Practice and applicable regulatory requirements and the AstraZeneca policy on bioethics. Informed consent was obtained from all patients prior to enrollment into the study. Data underlying the findings described in this manuscript may be obtained in accordance with AstraZeneca’s data-sharing policy described at http://astrazenecagrouptrials.pharmacm.com/ST/Submission/Disclosure. Full study protocol available at: https://astrazenecagrouptrials.pharmacm.com/ST/Submission/View?id=2318.

### Study design and participants

Full details of phase 3 randomized, open-label, international AURA3 study have been published previously^7^. In brief, AURA3 assessed the efficacy and safety of osimertinib versus platinum-pemetrexed chemotherapy in patients with centrally confirmed, *EGFR* T790M advanced NSCLC whose disease had progressed on first-line EGFR-TKI therapy. Patients were stratified (Asian/non-Asian) and randomized 2:1 to receive oral osimertinib (80 mg once daily) or intravenous chemotherapy (pemetrexed, 500 mg/m^2^ body-surface area) plus either cisplatin (75 mg/m^2^) or carboplatin (target area under the free carboplatin plasma concentration versus time curve of 5) every 3 weeks for up to six cycles, followed by optional pemetrexed maintenance therapy.

The analysis presented here was an exploratory, retrospective analysis to investigate candidate mechanisms of acquired resistance to osimertinib in the second-line treatment setting in a subset of patients who progressed or discontinued treatment during AURA3. Provision of ctDNA samples was mandatory for all patients who gave informed consent; samples from patients who withdrew consent were excluded. Evaluable patients were required to have detectable plasma EGFRm (L858R/ex19del) and/or *EGFR* T790M at baseline and to have paired plasma samples from baseline (day 1 cycle 1) and at progression and/or treatment discontinuation. Patients with non-detectable plasma EGFRm and/or *EGFR* T790M were excluded from the analysis. Plasma samples were restricted to those that passed quality control checks. In addition, patients from China were excluded as plasma samples were unable to be exported for analysis. The data-cutoff for this analysis was 15 March 2019, the final data-cut for AURA3 when OS was reported. Progression events were not updated at this cutoff.

Compulsory blood samples for plasma ctDNA were collected during the screening period. Serial plasma samples were collected (predose) from patients treated with osimertinib and platinum-pemetrexed at screening and on days 1, 8, and 15 of cycle 1 and day 1 of cycles 2–6, and then every 6 weeks thereafter until disease progression, and/or treatment discontinuation. Treatment beyond progression was permitted, and plasma samples were taken at both disease progression and treatment discontinuation. To explore mechanisms of acquired resistance, ctDNA extracted from paired plasma samples was analyzed using a 74-gene NGS panel (Guardant Health, Guardant360® assay)^34,35^. The limit of variant allelic fraction detected was 0.04–0.06%. Genomic alterations were identified using Guardant Health’s proprietary bioinformatics pipeline^34,35^. Paired samples were defined as samples from the same patient obtained on day 1 of the first cycle (baseline) and at progression or treatment discontinuation; where samples were available at both progression and or treatment discontinuation, data are reported based on the discontinuation sample.

### Assessments

Known and candidate-acquired resistance mechanisms were identified at progression and/or treatment discontinuation in both treatment arms, using the baseline plasma sample as a reference. Amplifications in *MET, HER2,* or *PIK3CA* were detected per GuardantHealth CLIA-validated protocols^34^.

PFS was assessed by the investigator according to response evaluation criteria in solid tumors (Response Evaluation Criteria in Solid Tumors [RECIST] 1.1). Tumor assessments were performed at baseline and every 6 weeks thereafter until objective disease progression. For the analysis reported here, duration of treatment, defined from the date of randomization to the end of osimertinib treatment, was determined according to candidate resistance mechanism in the osimertinib treatment arm.

### Statistical methods

As a retrospective, exploratory analysis, data were summarized using descriptive statistics. Plasma samples at progression or treatment discontinuation included in the paired analysis were collected up until April 2018. Clinical data were analyzed from 15 April 2016, data cutoff, and no further disease progression was assessed by RECIST after this date.

### Reporting summary

Further information on research design is available in the Nature Portfolio Reporting Summary linked to this article.

## Supplementary information

Description of Additional Supplementary Files

Supplementary Data 1

Reporting Summary

## Data Availability

The de-identified patient data generated in this study are provided in Supplementary Data 1. Specific consent for sequencing data deposition was not obtained from patients. Anonymized patient-level clinical data, aggregated clinical data, and/or anonymized clinical study documents underlying the findings described in this manuscript may be obtained in accordance with AstraZeneca’s data-sharing policy described at: http://astrazenecagrouptrials.pharmacm.com/ST/Submission/Disclosure. Since at the time of this publication, the AURA3 trial is still ongoing, the study data will be accessible at https://vivli.org/ when the trial is completed. In the meantime, requests to access the data from the AURA3 trial described in the current manuscript can be submitted through: https://vivli.org/members/enquiries-about-studies-not-listed-on-the-vivli-platform/. Requested data are available from approval of the request typically for one year. Some patients/countries may need to be excluded based on the informed consent form or country‐level legislation. The use of data must comply with the requirements of the Human Genetics Resources Administration of China and patients who have withdrawn consent for data use will be removed from the shared dataset. Patient-level images or genetic data are not available for access.
