# Supplementary material for: Analysis of acquired resistance mechanisms to osimertinib in patients with EGFR-mutated advanced non-small cell lung cancer from the AURA3 trial
Source: Nat Commun. 2023 Feb 27;14:1071. doi: 10.1038/s41467-023-35962-x (PMC9971022; doi:10.1038/s41467-023-35962-x)
Supplement: Supplementary file 1 — Description of Additional Supplementary Files [file 41467_2023_35962_MOESM1_ESM.pdf]

Description of Additional Supplementary Files

Supplementary Data 1: this excel file contains the raw, de-identified data required to enable a reader to reproduce the results presented in the figures.
