# Supplementary material for: Analysis of acquired resistance mechanisms to osimertinib in patients with EGFR-mutated advanced non-small cell lung cancer from the AURA3 trial
Source: Nat Commun. 2023 Feb 27;14:1071. doi: 10.1038/s41467-023-35962-x (PMC9971022; doi:10.1038/s41467-023-35962-x)
Supplement: Supplementary file 3 — Reporting Summary [file 41467_2023_35962_MOESM3_ESM.pdf]

nature portfolio

Corresponding author(s): Dr Vassiliki Papadimitrakopoulou

Last updated by author(s): Oct 12, 2022

Reporting Summary

Nature Portfolio wishes to improve the reproducibility of the work that we publish. This form provides structure for consistency and transparency in reporting. For further information on Nature Portfolio policies, see our Editorial Policies and the Editorial Policy Checklist.

Statistics

For all statistical analyses, confirm that the following items are present in the figure legend, table legend, main text, or Methods section.

n/a	Confirmed	
☐	☒	The exact sample size (n) for each experimental group/condition, given as a discrete number and unit of measurement
☐	☒	A statement on whether measurements were taken from distinct samples or whether the same sample was measured repeatedly
☒	☐	The statistical test(s) used AND whether they are one- or two-sided<br>*Only common tests should be described solely by name; describe more complex techniques in the Methods section.*
☒	☐	A description of all covariates tested
☒	☐	A description of any assumptions or corrections, such as tests of normality and adjustment for multiple comparisons
☐	☒	A full description of the statistical parameters including central tendency (e.g. means) or other basic estimates (e.g. regression coefficient) AND variation (e.g. standard deviation) or associated estimates of uncertainty (e.g. confidence intervals)
☐	☒	For null hypothesis testing, the test statistic (e.g. F, t, r) with confidence intervals, effect sizes, degrees of freedom and P value noted<br>*Give P values as exact values whenever suitable.*
☒	☐	For Bayesian analysis, information on the choice of priors and Markov chain Monte Carlo settings
☒	☐	For hierarchical and complex designs, identification of the appropriate level for tests and full reporting of outcomes
☒	☐	Estimates of effect sizes (e.g. Cohen's d, Pearson's r), indicating how they were calculated

Our web collection on statistics for biologists contains articles on many of the points above.

Software and code

Policy information about availability of computer code

Data collection	No software used
Data analysis	No software used

For manuscripts utilizing custom algorithms or software that are central to the research but not yet described in published literature, software must be made available to editors and reviewers. We strongly encourage code deposition in a community repository (e.g. GitHub). See the Nature Portfolio guidelines for submitting code & software for further information.

Data

Policy information about availability of data

All manuscripts must include a data availability statement. This statement should provide the following information, where applicable:

- Accession codes, unique identifiers, or web links for publicly available datasets
- A description of any restrictions on data availability
- For clinical datasets or third party data, please ensure that the statement adheres to our policy

The de-identified patient data generated in this study are provided in the Supplementary Information/Source Data file. Data underlying the findings described in this manuscript may be obtained in accordance with AstraZeneca's data sharing policy described at http://astrazenecagrouptrials.pharmacm.com/ST/Submission/Disclosure

Field-specific reporting

Please select the one below that is the best fit for your research. If you are not sure, read the appropriate sections before making your selection.

☒ Life sciences ☐ Behavioural & social sciences ☐ Ecological, evolutionary & environmental sciences

For a reference copy of the document with all sections, see nature.com/documents/nr-reporting-summary-flat.pdf

Life sciences study design

All studies must disclose on these points even when the disclosure is negative.

Sample size	No sample size calculation was performed for this exploratory analysis of resistance mechanisms but the initial sample size for the AURA3 study was determined as per that reported in the primary manuscript (Mok et al. NEJM 2017;376:629-640). From this publication, the sample size was determined based on 221 events of progression or death providing a power of 80% to reject the null hypothesis of no significant difference in the duration of progression-free survival between the two treatment groups, assuming a treatment effect hazard ratio of 0.67 with a P value of 0.05 indicating two-sided statistical significance.
Data exclusions	Only patients who had progressed or discontinued treatment were included in these resistance mechanisms analyses. Patients with a non-detectable plasma EGFR-TKI sensitizing mutation (EGFRm) and/or EGFR T790M mutation were excluded and only patients with paired plasma samples from baseline and at progression and/or treatment discontinuation were included. Patients were also excluded if they withdrew consent and patients from China were excluded as plasma samples were unable to be exported for analysis.
Replication	No specific measures were taken to verify the reproducibility of the findings. This manuscript and the accompanying FLAURA resistance sister manuscript demonstrate our attempt to validate the findings in a similar population and there are consistencies in the results (e.g. MET amplification and C797S/fusion mutations). We do not have samples from an identical AURA3-like population, so cannot directly validate some of the findings. However, FLAURA findings are being validated in ongoing studies, such as ELIOS and ORCHARD.
Randomization	Patients were stratified according to Asian or non-Asian race and were randomly assigned in a 2:1 ratio to receive osimertinib or platinum-pemetrexed chemotherapy.
Blinding	AURA3 was an open-label study so the investigators were not blinded to group allocation of the study treatment or to these exploratory analyses. Blinding of the original study is not especially relevant to this work, but the AURA3 study was open label because it is very challenging to blind an oral agent against a chemotherapy regimen. The resistance analysis reported in this manuscript was not blinded because we need to know the actual treatment patients received, in order to interpret resistance mutations - a mechanism of resistance to an unknown therapy is not useful for interpretation.

Reporting for specific materials, systems and methods

We require information from authors about some types of materials, experimental systems and methods used in many studies. Here, indicate whether each material, system or method listed is relevant to your study. If you are not sure if a list item applies to your research, read the appropriate section before selecting a response.

Materials & experimental systems

n/a	Involved in the study	
☒	☐	Antibodies
☒	☐	Eukaryotic cell lines
☒	☐	Palaeontology and archaeology
☒	☐	Animals and other organisms
☐	☒	Human research participants
☐	☒	Clinical data
☒	☐	Dual use research of concern

Methods

n/a	Involved in the study	
☒	☐	ChIP-seq
☒	☐	Flow cytometry
☒	☐	MRI-based neuroimaging

Human research participants

Policy information about studies involving human research participants

Population characteristics	Enrolled patients were adults (male or female; age range 25–85) with advanced non-small cell lung cancer with the EGFR T790M mutation who had progressed on first-line EGFR-tyrosine kinase therapy. Also as reported in Mok et al. NEJM 2017;376:629-640, patients with stable, asymptomatic central nervous system metastases that had not been treated with glucocorticoids for at least 4 weeks before the first dose of a trial drug were eligible for inclusion.
Recruitment	Eligible patients were recruited by investigators at study sites. Resistance analysis is limited to patients with detected ctDNA which is a known prognostic factor - we address this bias in the manuscript.
Ethics oversight	The study was approved by the institutional review board (IRB)/independent ethics committee

Ethics oversight	(IEC) associated with each study centre. For the IRB/IEC names and addresses, please request. Study protocol available: Full study protocol available at http://astrazenecagrouptrials.pharmacm.com/ST/Submission/Disclosure (noted on page 12 of the manuscript). This study was performed in accordance with the ethical principles that have their origin in the Declaration of Helsinki and that are consistent with International Conference on Harmonisation/Good Clinical Practice and applicable regulatory requirements and the AstraZeneca policy on bioethics. Informed consent was obtained from all patients prior to enrollment into the study.

Note that full information on the approval of the study protocol must also be provided in the manuscript.

Clinical data

Policy information about clinical studies

All manuscripts should comply with the ICMJE guidelines for publication of clinical research and a completed CONSORT checklist must be included with all submissions.

Clinical trial registration	NCT02151981
Study protocol	The study protocol was published along with the primary manuscript (Mok et al. NEJM 2017;376:629-640) and can be accessed at: https://www.nejm.org/doi/suppl/10.1056/NEJMoa1612674/suppl_file/nejmoa1612674_protocol.pdf
Data collection	Patients were recruited at 126 trial centers in Australia, Canada, China, France, Germany, Hong Kong, Hungary, Italy, Japan, Mexico, Netherlands, Russian Federation, South Korea, Spain, Sweden, Taiwan, the United Kingdom, and the United States of America (Mok et al. NEJM 2017;376:629-640). Clinical data were collected from August 2014 to September 201 and analyzed with a cut-off date of April 15, 2016 and in these exploratory analyses of resistance mechanisms, plasma samples at progression or treatment discontinuation included in the paired analysis were collected up until April 2018.
Outcomes	As this was an exploratory analysis of resistance mechanisms, no pre-defined primary or secondary outcome measures were assessed in this analysis. For these exploratory analyses, circulating tumor DNA (ctDNA) samples were assessed in patients who had progressed or discontinued treatment. Disease progression was assessed by the investigator, according to the Response Evaluation Criteria in Solid Tumors (RECIST) version 1.1, at baseline and every 6 weeks thereafter until objective progressive disease. Evaluable patients were required to have detectable plasma EGFRm (L858R/ex19del) and/or EGFR T790M at baseline and to have paired plasma samples from baseline (day 1 cycle 1) and at progression and/or treatment discontinuation. ctDNA samples were collected at screening and on days 1, 8 and 15 of cycle 1 and day 1 of cycles 2–6, and then every 6 weeks thereafter until disease progression, and/or treatment discontinuation. Acquired mechanisms of resistance were identified from ctDNA extracted from paired plasma samples (defined as samples from the same patient obtained on day 1 of the first cycle [baseline] and at progression or treatment discontinuation) analyzed using a 74 gene next-generation sequencing panel (Guardant Health, Guardant360® assay) or 500 gene panel (Guardant Health, GuardantOMNI assay). All 73 genes on the Guardant360 panel were included in the OMNI 500 gene panel. Known and candidate acquired resistance mechanisms were identified at progression and/or treatment discontinuation in both treatment arms, using the baseline plasma sample as a reference. Amplifications in MET, HER2 or PIK3CA were detected per GuardantHealth CLIA-validated protocol. For the analysis of duration of osimertinib treatment by candidate resistance mechanism, duration of treatment was defined as randomization to end of osimertinib treatment.
